# Angiotensin II Induces Cardiac Edema and Hypertrophic Remodeling through Lymphatic-Dependent Mechanisms

**DOI:** 10.1155/2022/5044046

**Published:** 2022-02-18

**Authors:** Jie Bai, Liangqingqing Yin, Wei-Jia Yu, Yun-Long Zhang, Qiu-Yue Lin, Hui-Hua Li

**Affiliations:** ^1^Institute of Cardiovascular Diseases, First Affiliated Hospital of Dalian Medical University, No. 193, Lianhe Road, Xigang District, Dalian 11600, China; ^2^Department of Emergency Medicine, Beijing Key Laboratory of Cardiopulmonary Cerebral Resuscitation, Beijing Chaoyang Hospital, Capital Medical University, Beijing 100020, China

## Abstract

Cardiac lymphatic vessel growth (lymphangiogenesis) and integrity play an essential role in maintaining tissue fluid balance. Inhibition of lymphatic lymphangiogenesis is involved in cardiac edema and cardiac remodeling after ischemic injury or pressure overload. However, whether lymphatic vessel integrity is disrupted during angiotensin II- (Ang II-) induced cardiac remodeling remains to be investigated. In this study, cardiac remodeling models were established by Ang II (1000 ng/kg/min) in VEGFR-3 knockdown (Lyve-1^Cre^ VEGFR-3^f/−^) and wild-type (VEGFR-3^f/f^) littermates. Our results indicated that Ang II infusion not only induced cardiac lymphangiogenesis and upregulation of VEGF-C and VEGFR-3 expression in the time-dependent manner but also enhanced proteasome activity, MKP5 and VE-cadherin degradation, p38 MAPK activation, and lymphatic vessel hyperpermeability. Moreover, VEGFR-3 knockdown significantly inhibited cardiac lymphangiogenesis in mice, resulting in exacerbation of tissue edema, hypertrophy, fibrosis superoxide production, inflammation, and heart failure (HF). Conversely, administration of epoxomicin (a selective proteasome inhibitor) markedly mitigated Ang II-induced cardiac edema, remodeling, and dysfunction; upregulated MKP5 and VE-cadherin expression; inactivated p38 MAPK; and reduced lymphatic vessel hyperpermeability in WT mice, indicating that inhibition of proteasome activity is required to maintain lymphatic endothelial cell (LEC) integrity. Our results show that both cardiac lymphangiogenesis and lymphatic barrier hyperpermeability are implicated in Ang II-induced adaptive hypertrophic remodeling and dysfunction. Proteasome-mediated hyperpermeability of LEC junctions plays a predominant role in the development of cardiac remodeling. Selective stimulation of lymphangiogenesis or inhibition of proteasome activity may be a potential therapeutic option for treating hypertension-induced cardiac remodeling.

## 1. Introduction

Cardiac remodeling is generally accepted as a determinant of heart failure (HF), a major contributor to morbidity and mortality worldwide. This dynamic process includes many functional and structural alterations, such as cardiac contractile dysfunction, hypertrophy, interstitial fibrosis, and myocyte apoptosis [1]. Increasing evidence demonstrates that multiple factors modulate the pathogenesis of cardiac remodeling, including angiotensin II (Ang II), high salt intake, inflammation, and oxidative stress, which activate multiple molecular pathways, including the AKT/mTOR, MEK/ERK, JAK/STAT, and TGF-*β*/Smad pathways, thereby resulting in cardiac remodeling and dysfunction [1]. Previous investigations exploring the pathophysiological mechanisms of cardiac remodeling have primarily concentrated on the vasculature, myocardial cells, or myofibroblasts [2, 3]. The roles of lymphatic growth (lymphangiogenesis) and function in the regulation of ischemia and pressure overload of the heart have also been identified [4]. However, the involvement of lymphatic vessel growth and integrity in Ang II-induced cardiac remodeling is still unknown.

The mammalian heart has a largely intricate lymphatic system that plays a crucial role in maintaining tissue fluid balance [5]. Lymphatic endothelial cells (LECs), which are shaped like oak leaf leaves, form blind-ended lymphatic capillaries, which are tightly and loosely connected to each other by a series of nonconsecutive adhesion junctions that control fluid and cell outflow [6–8]. According to a report, VEGF-C and its receptor VEGFR-3 act as major signals for lymphatic growth [9]. Inhibition of cardiac lymphangiogenesis leads to various cardiovascular disorders, including atherosclerosis, myocardial infarction, and HF. Conversely, selective activation of lymphangiogenesis in the heart has a cardioprotective effect [4]. Recently, our study demonstrated that Ang II stimulates the proliferation and migration of LECs via the Ang II type 1 receptor [10]. Moreover, LECs are joined by specialized cell–cell junctions to maintain lymphatic vessel integrity, which is required for maintaining tissue liquid homeostasis [11]. Furthermore, vascular endothelial cadherin (VE-cadherin) is important in maintaining the homeostasis of endothelial adherent junctions and lymphatic vessel permeability [12, 13]. Interestingly, several studies have demonstrated that lymphatic vessel leakage and hyperpermeability are involved in different diseases, such as cancer metastasis, atherosclerosis, and type 2 diabetes [14–16]. However, whether similar lymphatic dysfunction occurs during Ang II-induced cardiac remodeling has not been investigated.

In our current study, by stimulating VEGFR-3 knockdown (Lyve-1^Cre^ VEGFR-3^f/−^) and wild-type (WT) mice and mouse LECs stimulated with a selective proteasome inhibitor (epoxomicin), we found that Ang II caused cardiac edema and hypertrophic remodeling accompanied by increased lymphangiogenesis and hyperpermeability in a time-dependent manner. Moreover, VEGFR-3 knockdown in mice inhibited lymphangiogenesis resulting in deterioration of cardiac edema, hypertrophic remodeling, and contractile dysfunction after Ang II infusion. Conversely, administration of epoxomicin (a selective proteasome inhibitor) to WT mice significantly suppressed Ang II-induced cardiac lymphatic hyperpermeability and edema, resulting in amelioration of cardiac remodeling, suggesting that proteasome-mediated lymphatic vessel hyperpermeability plays a predominant role in disease progression. Therefore, selective stimulation of lymphangiogenesis or suppression of proteasome activity may be a potential therapeutic option for hypertension-induced cardiac remodeling.

## 2. Materials and Methods

### 2.1. Animal Models

Lyve-1^Cre^ [17] and VEGFR-3-floxed (VEGFR-3^f/f^) mice were obtained from the Jackson Laboratory and the European Mouse Mutant Archive, respectively. In these mice, the Lyve-1 promoter activates Cre recombinase in LECs, and the VEGFR-3-floxed fragment will be deleted when it combines with a Cre recombinase. To globally delete the VEGFR-3 gene in LECs of the mouse, the homozygous Lyve-1^Cre^ mice were mated with the homozygous VEGFR-3^f/f^ mice to generate Lyve-1 heterozygous and VEGFR-3 heterozygous genotype mice (referred to as F1 generation), then the F1 generation mice were secondly mated with the homozygous Lyve-1^Cre^ mice to generate Lyve-1 homozygous and VEGFR-3 heterozygous genotype mice (referred to as F2 generation, Lyve-1^Cre^ VEGFR-3^f/−^). The Lyve-1^Cre^ VEGFR-3^f/−^ mice were used in the current study because VEGFR-3 homozygosity was embryonic lethal [18, 19] and used the VEGFR-3^f/f^ mice as controls. Cardiac remodeling was induced by infusion of Ang II (1000 ng/kg/min) for 3-14 days as previously described [20]. The mice were injected i.p. with control (PBS) or epoxomicin (0.58 mg/kg/day, Selleck, S7038) before Ang II or saline infusion as described in a previous report [21]. The mice were housed in a specific pathogen-free environment, and the experimental protocols were approved by the Animal Care and Use Committee of Dalian Medical University (Number: AEE20027).

### 2.2. Blood Pressure Measurement and Echocardiography

The average systolic blood pressure (SBP) in the aorta was monitored using tail-cuff equipment as previously described [22]. Cardiac functions were evaluated by an echocardiographic instrument in M-mode (Vevo 1100 System, Canada) during anesthesia with 1.5% isoflurane. Echocardiographic parameters were measured and calculated as previously described [19, 20, 22].

### 2.3. Histological and Immunohistochemical Staining

The animals were killed with an overdose of isoflurane (>5%). Their hearts were dehydrated and embedded after fixation with 4% paraformaldehyde. Paraffin sections were subjected to hematoxylin and eosin (H&E) staining and Masson's trichrome stain (G1340-7, Beijing Solarbio Science & Technology Co., Ltd.). Heart sections were subjected to immunohistochemical staining with an alpha-smooth muscle actin antibody (*α*-SMA, ARG66381), and then, development was performed with DAB as previously described [19].

### 2.4. Immunofluorescence Assay

Heart tissues were fixed, dehydrated in 20% sucrose, and then embedded in optimal cutting temperature compound. Wheat germ agglutinin (WGA) staining was performed to estimate cardiomyocyte (CM) hypertrophy. For immunofluorescence, heart sections or cells were incubated with an anti-CD68 (ab125212, 1 : 200), anti-VEGFR-3 (ab273148, 1 : 200), anti-LYVE-1 (NOVUS, NBP1-43411, 1 : 200), or anti-VE-cadherin (ab205336, 1 : 200) antibody and then with fluorescently labeled antibodies (Life-Lab, 1 : 200, CN). DHE staining was performed to measure the reactive oxygen species (ROS) levels as previously described [23]. To quantify staining intensity, four sections from areas at least 60 mm apart in each heart were randomly selected. At least six nonoverlapping fields of each section were imaged with a fluorescence microscope.

### 2.5. Cardiac Water Content Analysis

Whole hearts were isolated, and the wet weight was obtained. Then, the tissues were dried at 65°C for 5 days to obtain the dry weight. The cardiac water content was calculated as previously described [19].

### 2.6. Cell Experiments

LECs were isolated in our laboratory and cultured as previously described [10]. For *in vitro* assays, the LECs were pretreated with losartan (MedChemExpress, MCE, 10 *μ*M) and epoxomicin (10 *μ*M) and treated with saline or Ang II (100 nM) for 24 hours.

### 2.7. In Vitro and In Vivo Permeability Assays

Two different methods were used to assess the permeability of LECs *in vitro*. (1) First, 100 *μ*L of Evans blue (0.67 mg/ml) was added to the upper chamber, and 600 *μ*L of PBS was added to the lower chamber. Then, 100 *μ*L aliquots were obtained from the lower compartment and analyzed at 0.5, 1, 2, and 3 hours. The OD value (620 nm) was measured with a continuous wavelength microplate reader (TECAN, CA). (2) Next, 100 *μ*L of FITC-dextran (1 mg/mL) was added to the migration medium in the top compartment, and 600 *μ*L of PBS was added to the lower chamber. Then, 100 *μ*L aliquots were obtained from the lower compartment and analyzed with a microplate reader at 0.5, 1, 2, and 3 hours [24, 25]. For *in vivo* assays, we injected all the mice with Evans blue (0.67 mg/mL) into the footpad popliteal lymph nodes. After 16 hours, we removed the lymph nodes and ground them in 100 *μ*L of PBS, and then, the OD value (620 nm) was measured with a continuous wavelength microplate reader [24].

### 2.8. Real-Time PCR

Total RNA was purified from fresh hearts or cells with TRIzol (Sangon Biotech, B511311). cDNA (1 *μ*g) was obtained with PrimeScript RT reagent (Yeasen, 11141ES60). A SYBR Green Premix Pro Taq HS qPCR Kit (Accurate Biotechnology Co., Ltd., Hunan, AG11701) was used to perform quantitative real-time PCR on the 7500 Fast system (ABI, US). The results were analyzed using the *ΔΔ*Ct method, and mRNA levels were normalized to the content of GAPDH. The primer sequences are listed in Table S1.

### 2.9. Immunoblotting

Total protein was obtained from snap-frozen hearts or cells using a protein extraction kit (Keygenbio, KGP250) and a 1.5 mL tube (Guangzhou Jet Bio-Filtration Co., Ltd.). Thirty micrograms of protein was separated by SDS–PAGE and then transferred onto PVDF membranes. The membranes were incubated at 4°C overnight with conjugated antibodies (Sino Biological Inc., 1 : 2000). The immunoblotting bands were detected by chemiluminescence. The following antibodies were used: anti-VEGF-C (22601-1-AP, Proteintech), anti-VEGFR-3 (ab273148, Abcam), anti-p-p38 (ARG51850, Arigo), anti-p38 (ARG55258, Arigo), anti-VE-cadherin (ab205336, Abcam), anti-p-ERK1/2 (4370, CST), anti-ERK1/2 (4695S, CST), anti-p-AKT (9271S, CST), anti-AKT (9272, CST), anticalcineurin A (CaNA) (2614, CST), anti-p-STAT3 (9145, CST), anti-STAT3 (9139, CST), anti-TGF-*β*1 (3711, CST), anti-p-Smad2/3 (882S, CST), anti-Smad2/3 (8685, CST), anti-NOX2 (19013-1-AP, Proteintech), anti-NOX4 (14347-1-AP, Proteintech), anti-p-P65 (3033, CST), anti-P65 (4764, CST), anti-*β*2i (13635, CST), anti-*β*5i (ARG41022, Arigo) anti-MKP5 (374276, Santa), and anti-GAPDH (5174, CST). ImageJ software was used for densitometry analysis.

### 2.10. Measurement of Proteasome Activity

Protein was extracted from heart tissues with HEPES buffer as previously described [26], and the protein concentration was quantified with a BCA Kit (US EVERBRIGHT, W6006). Twenty micrograms of total protein from each group was mixed with HEPES to achieve a total volume of 50 *μ*L. All the samples, reagents, and buffers were added to 96-well plates for measurement of the levels of *β*1i, *β*2i, and *β*5i. The fluorescence intensity was measured using a continuous wavelength microplate reader as previously described [27].

### 2.11. Enzyme-Linked Immunosorbent Assay

Serum VEGF-C concentrations were measured with an enzyme-linked immunosorbent assay kit (Boster) according to the manufacturer's instructions. The OD values were measured at 450 nm.

### 2.12. Statistical Analyses

The data are shown as the mean ± standard deviation. GraphPad Prism 9 was used to analyze the significance of the differences. Independent *t* test was used to analyze differences between two groups when the data were normally distributed, and the Mann–Whitney test was used for nonnormally distributed data. One- or two-way ANOVA was used to analyze differences among multiple groups. *p* < 0.05 was defined as statistically significant.

## 3. Results

### 3.1. Ang II Infusion Induces Cardiac Remodeling, Lymphangiogenesis, and Endothelial Hyperpermeability

**Figure 1:**
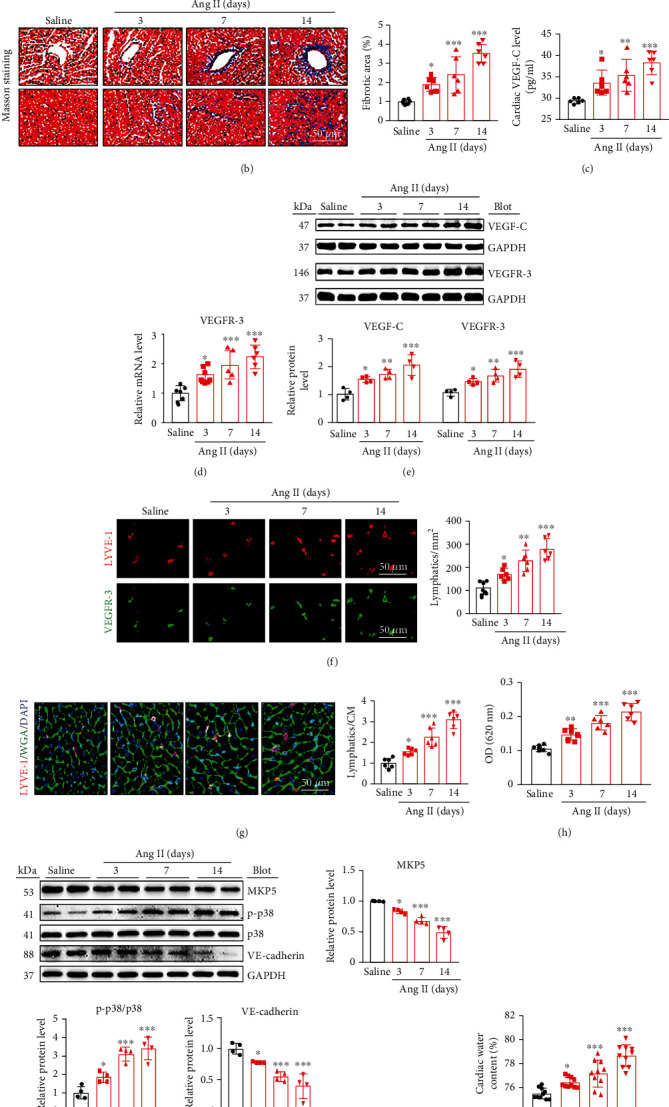
Dynamic changes in cardiac hypertrophy, fibrosis, lymphangiogenesis, lymphatic permeability, and water content in Ang II-infused mice. WT mice were infused with Ang II (1000 ng/kg/min) or saline for 3, 7, and 14 days. (a) H&E staining (left) and the HW/TL ratio (right, *n* = 6). (b) Masson staining (left) and quantitative analysis of the extent of myocardial fibrosis (%) (right, *n* = 6). (c) Measurement of mouse serum VEGF-C concentrations (*n* = 6). (d) qPCR analysis of cardiac VEGFR-3 mRNA expression (*n* = 6). (e) Immunoblot analysis of cardiac VEGF-C and VEGFR-3 levels (left) and quantification (right, *n* = 4). (f) Immunostaining with antibodies against LYVE-1 (red) and VEGFR-3 (green) (left) and quantification of cardiac lymphatics (right, *n* = 6). (g) Cardiac staining with an LYVE-1 antibody (red), WGA (green), and DAPI (blue) (left), and the ratio of cardiac lymphatics to CMs (right, *n* = 6). (h) Evaluation of permeability *in vivo* 16 hours after footpad injection of Evans blue (0.67 mg/mL) into the popliteal lymph nodes (*n* = 6). (i) Immunoblot analysis of cardiac MKP5, p-p38, p38, VE-cadherin, and GAPDH levels and quantification of the levels of these proteins (*n* = 4). (j) Quantitative analysis of cardiac edema (*n* = 10). The data are presented as the mean ± SD; *n* represents the number of animals in each group. Statistical differences were determined by one-way ANOVA; ^∗^*p* < 0.05, ^∗∗^*p* < 0.01, and ^∗∗∗^*p* < 0.001 versus the saline group.

To evaluate the potential role of cardiac lymphatics, we infused mice with Ang II to induce pathological cardiac remodeling. The average SBP progressively increased after Ang II infusion for 14 days (Figure S1(a)). Cardiac hypertrophy and fibrosis, as indicated by increases in heart size, the heart weight to body weight ratio (HW/TL), and fibrotic area, increased in a time-dependent manner in Ang II-infused mice compared with saline-treated controls (Figures 1(a) and 1(b)). In agreement with these pathological changes, the mRNA levels of hypertrophic markers (ANF and BNP) and fibrotic markers (collagen I, collagen III, and *α*-SMA) were increased in a time-dependent manner after Ang II infusion compared with saline infusion (Figures S1(b)–1(d)).

Since lymphangiogenesis plays a significant role in regulating cardiac edema and remodeling induced by ischemia/reperfusion injury and pressure overload [19], we then investigated whether Ang II influences cardiac lymphangiogenesis during this process. Consistent with our *in vitro* findings [10], the blood VEGF-C concentration and VEGFR-3 mRNA levels were increased in a time-dependent manner in the Ang II-infused mice compared with saline-treated controls (Figures 1(c) and 1(d)). The increases in VEGF-C and VEGFR-3 protein levels in the hearts of Ang II-infused mice were further confirmed by immunoblotting (Figure 1(e)). Accordingly, cardiac lymphangiogenesis, as indicated by increases in the number of LYVE-1and VEGFR-3 double-positive lymphatic vessels and the ratio of LYVE-1-positive vessels to CMs, was markedly enhanced during Ang II infusion (Figures 1(f) and 1(g)), suggesting that cardiac lymphangiogenesis is increased during Ang II-induced cardiac remodeling.

Leakage of LECs is crucial for tissue edema formation [28]. We next evaluated lymphatic vessel permeability *in vivo* after footpad injection of Evans blue into the popliteal lymph nodes at 16 hours. Compared with saline infusion, Ang II infusion markedly increased lymphatic vessel permeability, as reflected by an increased OD (620 nm) (Figure 1(h)). Furthermore, we examined the protein levels of MKP5 (a negative regulator of p38), p38, and VE-cadherin, which are related to key signaling pathways involved in LEC permeability. Immunoblotting revealed that the protein expression of p-p38 was upregulated whereas the protein expression of MKP5 and VE-cadherin was downregulated after Ang II infusion compared with saline infusion (Figure 1(i)). Then, we assessed cardiac water content by the wet-dry weight method and found that Ang II infusion increased cardiac water content in a time-dependent manner in mice (Figure 1(j)), indicating that Ang II infusion successfully induced hyperpermeability of LECs, leading to cardiac edema. Together, these data showed that Ang II infusion leads to an imbalance of cardiac lymphangiogenesis and lymphatic hyperpermeability, which may result in Ang II-induced cardiac edema and remodeling.

### 3.2. VEGFR-3 Knockdown Reduces Cardiac Lymphangiogenesis, Leading to Cardiac Edema

**Figure 2:**
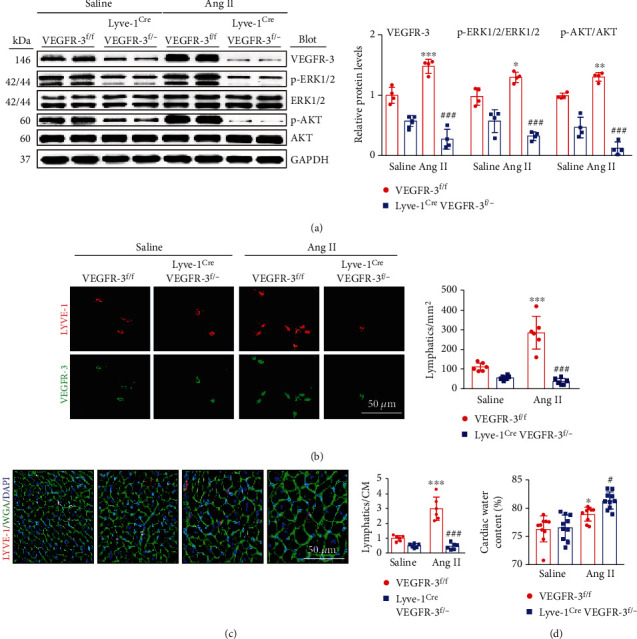
VEGFR-3 knockdown suppresses Ang II-induced cardiac lymphangiogenesis. VEGFR-3^f/f^ and Lyve-1^Cre^ VEGFR-3^f/−^ mice were treated with saline or Ang II (1000 ng/kg/min) for 14 days. (a) Immunoblot analysis of cardiac VEGFR-3, p-ERK1/2, ERK1/2, p-AKT, and AKT levels (left) and quantification (right, *n* = 4). (b) Cardiac staining with LYVE-1 (red) and VEGFR-3 (green) antibodies (left) and quantification of cardiac lymphatics (right, *n* = 6). (c) Cardiac staining with an LYVE-1 antibody (red), WGA (green), and DAPI (blue) (left) and quantification of the ratio of cardiac lymphatics to CM (right, *n* = 6). (d) Quantitative analysis of cardiac edema (*n* = 10). The data are presented as the mean ± SD; *n* represents the number of animals in each group. Statistical differences were determined by two-way ANOVA; ^∗^*p* < 0.05, ^∗∗^*p* < 0.01, and ^∗∗∗^*p* < 0.001 versus the VEGFR-3^f/f^+saline group; ^#^*p* < 0.05 and ^###^*p* < 0.001 versus the VEGFR-3^f/f^+Ang II group.

VEGFR-3 is key for the regulation of cardiac lymphangiogenesis in hearts exposed to ischemia and pressure overload [19]. To determine whether lymphangiogenesis is involved in Ang II-induced cardiac hypertrophic remodeling, we first generated VEGFR-3 knockdown (Lyve-1^Cre^ VEGFR-3^f/−^) mice and littermates (VEGFR-3^f/f^). Consistent with our previous findings [19], immunoblotting revealed that the expression of VEGFR-3 and its downstream molecules (p-AKT and p-ERK1/2) in Lyve-1^Cre^ VEGFR-3^f/−^ mice was significantly lower than that in VEGFR-3^f/f^ mice (Figure 2(a)), indicating that VEGFR-3 knockdown inhibits Ang II-induced activation of AKT and ERK signals in the heart. Then, we evaluated the effect of VEGFR-3 on cardiac lymphangiogenesis. Immunostaining indicated that the Ang II-induced increases in the number of LYVE-1 and VEGFR-3 double-positive lymphatics and the ratio of LYVE-1-positive vessels to CMs observed in VEGFR-3^f/f^ mice were markedly reduced in Lyve-1^Cre^ VEGFR-3^f/−^ mice after Ang II infusion (Figures 2(b) and 2(c)). Accordingly, we measured the cardiac water content, and the data indicated that Ang II infusion markedly exacerbated cardiac edema in VEGFR-3^f/f^ mice and that this effect was further amplified in Ang II-infused Lyve-1^Cre^ VEGFR-3^f/−^ mice (Figure 2(d)). Therefore, these data showed that VEGFR-3 knockdown inhibits Ang II-induced cardiac lymphangiogenesis, resulting in exacerbation of cardiac edema.

### 3.3. VEGFR-3 Knockdown Exacerbates Ang II-Induced Cardiac Dysfunction and Hypertrophy

**Figure 3:**
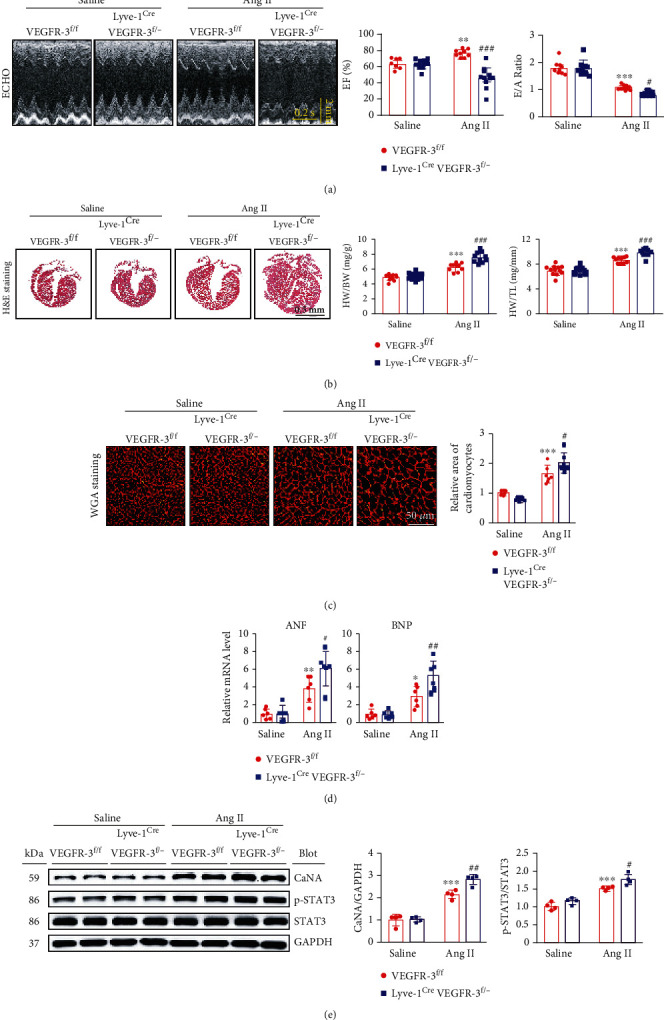
VEGFR-3 knockdown aggravates Ang II-induced cardiac dysfunction and hypertrophy in mice. VEGFR-3^f/f^ and Lyve-1^Cre^ VEGFR-3^f/−^ mice were infused with saline or Ang II (1000 ng/kg/min) for 14 days. (a) Echocardiography of the heart (left), left ventricular (LV) EF%, and the E/A ratio (right, *n* = 10). (b) Cardiac H&E staining (left) and the HW/BW and HW/TL ratio (right, *n* = 10). (c) Cardiac WGA staining (left) and quantitative analysis (right, *n* = 6). (d) qPCR analysis of cardiac ANF and BNP levels (*n* = 6). (e) Immunoblot analysis of cardiac CaNA, p-STAT3, and STAT3 levels (left) and quantification (right, *n* = 4). The data are presented as the mean ± SD; *n* represents the number of animals in each group. Statistical differences were determined by two-way ANOVA; ^∗^*p* < 0.05, ^∗∗^*p* < 0.01, and ^∗∗∗^*p* < 0.001 versus the VEGFR-3^f/f^+saline group; ^#^*p* < 0.05, ^##^*p* < 0.01, and ^###^*p* < 0.001 versus the VEGFR-3^f/f^+Ang II group.

We next tested whether VEGFR-3 influences Ang II-induced cardiac remodeling in VEGFR-3^f/f^ and Lyve-1^Cre^ VEGFR-3^f/−^ mice. Following 14 days of Ang II treatment, the elevation of average SBP observed in VEGFR-3^f/f^ mice was significantly enhanced in Lyve-1^Cre^ VEGFR-3^f/−^ mice (Figure S2). Moreover, VEGFR-3^f/f^ mice displayed features of cardiac dysfunction, as indicated by an obvious increase in ejection fraction (EF%) and a reduction in the peak early transmitral inflow mitral E velocity to transmitral inflow velocity due to atrial contraction (E/A) ratio; however, Lyve-1^Cre^ VEGFR-3^f/−^ mice exhibited decreased contractility and diastolic performance after Ang II infusion (Figure 3(a), Table S2). Furthermore, Ang II-induced cardiac hypertrophic remodeling, as indicated by increases in heart size (Figure 3(b)), the HW/BW and HW/TL ratios (Figure 3(b)), the cross-sectional area of CMs (Figure 3(c)) and ANF and BNP mRNA levels (Figure 3(d)), and upregulation of CaNA, and p-STAT3 protein expression in VEGFR-3^f/f^ mice was markedly accelerated in Lyve-1^Cre^ VEGFR-3^f/−^ mice (Figure 3(e)).

### 3.4. VEGFR-3 Knockdown Enhances Ang II-Induced Myocardial Fibrosis, Oxidative Stress, and Inflammation

**Figure 4:**
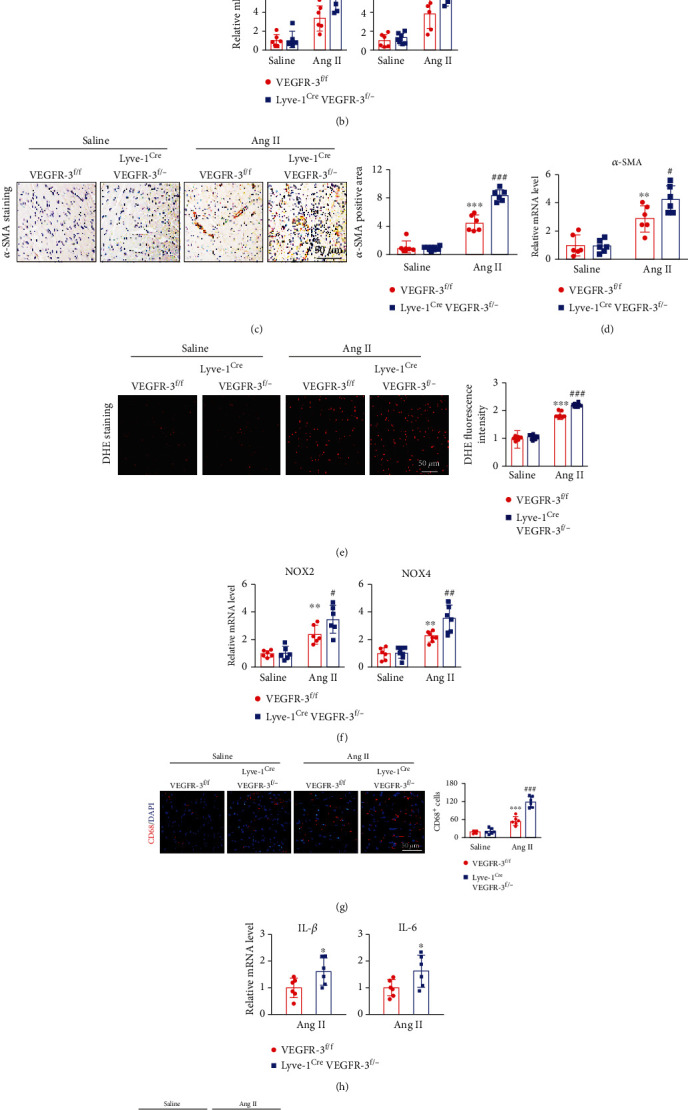
VEGFR-3 knockdown accelerates Ang II-induced cardiac fibrosis, oxidative stress, and inflammation in mice. VEGFR-3^f/f^ and Lyve-1^Cre^ VEGFR-3^f/−^ mice were infused with saline or Ang II (1000 ng/kg/min) for 14 days. (a) Cardiac Masson staining (left) and quantitative analysis of the fibrotic area (%) (right, *n* = 6). (b) qPCR analyses of myocardial collagen I and collagen III levels (*n* = 6). (c) Cardiac *α*-SMA immunohistochemical staining (left) and quantitative analysis of the positive area (right, *n* = 6). (d) qPCR analyses of myocardial *α*-SMA levels (*n* = 6). (e) Cardiac DHE fluorescence staining (left) and quantitative analysis of relative fluorescence intensity (right, *n* = 6). (f) qPCR analyses of myocardial NOX2 and NOX4 levels (*n* = 6). (g) CD68 (red) and DAPI (blue) fluorescence staining of heart sections (left) and quantification of CD68^+^ macrophages (right, *n* = 6). (h) qPCR analyses of myocardial IL-1*β* and IL-6 levels (*n* = 6). (i) Immunoblot analysis of cardiac TGF-*β*1, p-Smad2/3, Smad2/3, NOX2, NOX4, p-P65, and P65 levels (left) and quantification (right, *n* = 4). The data are presented as the mean ± SD; *n* represents the number of animals in each group. Statistical differences were determined by two-way ANOVA; ^∗^*p* < 0.05, ^∗∗^*p* < 0.01, and ^∗∗∗^*p* < 0.001 versus the VEGFR-3^f/f^+saline group; ^#^*p* < 0.05, ^##^*p* < 0.01, and ^###^*p* < 0.001 versus the VEGFR-3^f/f^+Ang II group.

Subsequently, we evaluated the influence of VEGFR-3 knockdown on cardiac fibrosis, oxidative stress, and inflammation after Ang II infusion. Compared with saline treatment, Ang II markedly increased myocardial collagen deposition and the number of ɑ-SMA^+^ myofibroblasts in VEGFR-3^f/f^ mice, as manifested by increases in the fibrotic area (%); the *α*-SMA^+^ area; and collagen I, collagen II, and *α*-SMA mRNA levels. These pathological responses were more obvious in Lyve-1^Cre^ VEGFR-3^f/−^ mice infused with Ang II (Figures 4(a)–4(d)). Accordingly, superoxide production, as reflected by DHE fluorescence intensity, and the mRNA levels of the NADPH oxidase isoforms NOX2 and NOX4 were significantly higher in Lyve-1^Cre^ VEGFR-3^f/−^ mice than in VEGFR-3^f/f^ mice after Ang II infusion (Figures 4(e) and 4(f)). Furthermore, the Ang II-induced increases in the number of CD68^+^ macrophages and the mRNA levels of the proinflammatory cytokines IL-1*β* and IL-6 observed in VEGFR-3^f/f^ mice were markedly amplified in Lyve-1^Cre^ VEGFR-3^f/−^ mice (Figures 4(g) and 4(h)). In agreement with these pathological changes, the protein expression of fibrotic, oxidative, and proinflammatory signaling mediators, including TGF-*β*1, p-Smad2/3, NOX2, NOX4, and p-P65, was more upregulated in Lyve-1^Cre^ VEGFR-3^f/−^ mice after Ang II treatment (Figure 4(i)). Overall, these data indicated that VEGFR-3-mediated cardiac lymphangiogenesis is essential for the inhibition of cardiac edema and remodeling.

### 3.5. Ang II Enhances Cardiac Proteasome Activity and the Degradation of Permeability-Related Proteins in the Heart and in Cultured LECs

**Figure 5:**
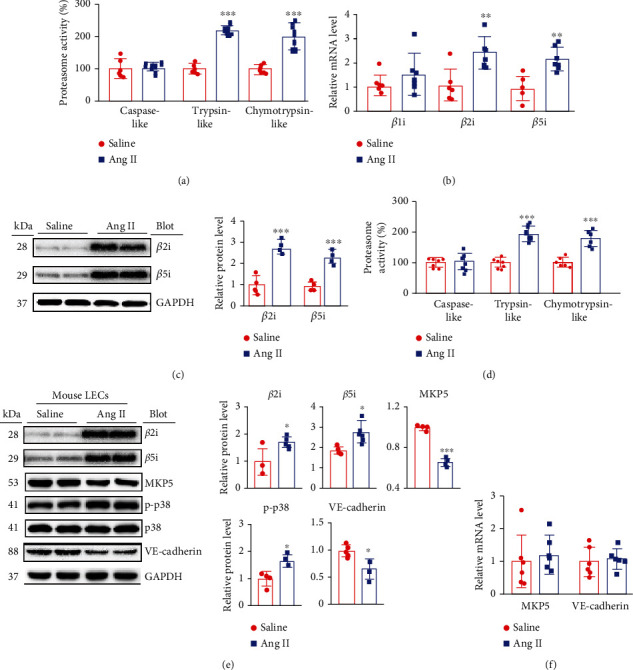
Ang II increases proteasome activity and immunoproteasome catalytic subunit levels in mice and cultured LECs. (a) The proteasome activity (%) of caspase-like, trypsin-like, and chymotrypsin-like proteins in the heart (*n* = 6). (b) qPCR analyses of myocardial *β*1i, *β*2i, and *β*5i levels (*n* = 6). (c) Immunoblot analysis of *β*2i and *β*5i levels (left) and quantification (right, *n* = 4). (d) LECs were stimulated with saline or Ang II (100 nM) for 24 hours. The proteasome activity (%) of caspase-like, trypsin-like, and chymotrypsin-like proteins in the heart (*n* = 6). (e) Immunoblot analysis of *β*2i, *β*5i, MKP5, p-p38, p38, and VE-cadherin levels (left) and quantification (right, *n* = 4). (f) qPCR analyses of MKP5 and VE-cadherin levels in LECs (*n* = 6). The data are presented as the mean ± SD; *n* represents the number of animals in or experiments for each group. Statistical differences were determined by *t* tests; ^∗^*p* < 0.05, ^∗∗^*p* < 0.01, and ^∗∗∗^*p* < 0.001 versus the saline group.

Based on our previous data that Ang II infusion markedly increases cardiac proteasome activity and immunoproteasome subunit expression in mice [26, 29] and our current results showing that MKP-5, VE-cadherin, and p38 MAPK might be important signaling pathways that induce lymphatic hyperpermeability leading to cardiac edema (Figures 1(h)–1(j)) and that these proteins are regulated by the proteasome [30, 31], we assessed whether Ang II-induced activation of the proteasome can cause degradation of the proteins MKP-5 and VE-cadherin and activation of p38 MAPK in the heart and LECs *in vitro*. Consistent with previous data, compared with saline infusion, Ang II infusion mainly increased trypsin-like and chymotrypsin-like activity and the expression of the immunoproteasome catalytic subunits *β*2i and *β*5i at both the mRNA and protein levels in the heart (Figures 5(a)–5(c)).

To confirm the specific effect of Ang II on the expression of *β*2i and *β*5i in mouse LECs, LECs were infused with Ang II for 24 hours. Compared with saline, Ang II resulted in significant increases in proteasome trypsin-like and chymotrypsin-like activity (Figure 5(d)) and the expression levels of *β*2i, *β*5i, and p-p38 MAPK and marked decreases in the protein levels of MKP5 and VE-cadherin in LECs (Figure 5(e)). However, the mRNA levels of MKP5 and VE-cadherin were not changed in LECs after Ang II stimulation (Figure 5(f)). These data showed that Ang II increases proteasome activity, which may be involved in the degradation of both MKP5 and VE-cadherin and that activation of p38 MAPK leads to LEC hyperpermeability.

### 3.6. Blockage of Proteasome Activity Reverses Ang II-Induced Hyperpermeability of LECs In Vitro

**Figure 6:**
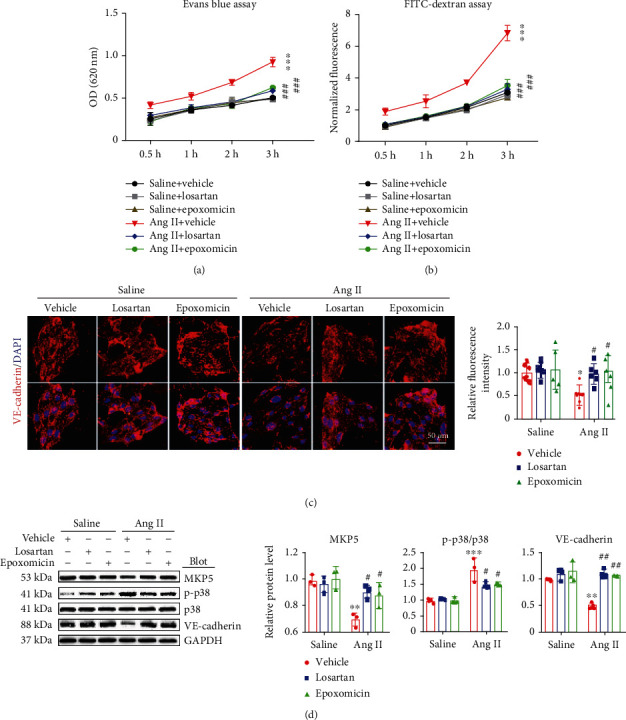
Suppression of proteasome activity alleviates Ang II-stimulated LEC hyperpermeability *in vitro*. LECs on the upper surface of the Transwell membrane were pretreated with vehicle, losartan (10 *μ*M), epoxomicin (10 *μ*M), or vehicle control and then treated with saline or Ang II (100 nM) for 24 hours. (a) Permeability was analyzed 0.5, 1, 2, and 3 hours after 100 *μ*L of Evans blue (0.67 mg/mL) was added to the upper compartment and 600 *μ*L of PBS was added to the lower compartment (*n* = 3). (b) Permeability was analyzed 0.5, 1, 2, and 3 hours after 100 *μ*L of FITC-dextran (1 mg/mL) was added to the upper compartment and 600 *μ*L of PBS was added to the lower compartment (*n* = 3). (c) VE-cadherin (red) and DAPI (blue) staining of LECs (left) and quantification (right, *n* = 6). (d) Immunoblot analysis of MKP5, p-p38, p38, and VE-cadherin levels (left) and quantification (right, *n* = 3). The data are presented as the mean ± SD; *n* represents the number of experiments for each group. Statistical differences were determined by two-way ANOVA; ^∗^*p* < 0.05, ^∗∗^*p* < 0.01, and ^∗∗∗^*p* < 0.001 versus the saline+vehicle group; ^#^*p* < 0.05, ^##^*p* < 0.01, and ^###^*p* < 0.001 versus the Ang II+vehicle group.

To test whether proteasome activity regulates Ang II-induced LEC hyperpermeability *in vitro*, we first performed leakage experiments with Evans's blue and FITC-conjugated dextran to examine LEC permeability. Compared with saline, Ang II significantly increased the hyperpermeability of LECs, whereas this effect was fully inhibited by losartan (an angiotensin type I receptor antagonist) and epoxomicin (a selective proteasome inhibitor) (Figures 6(a) and 6(b)). Moreover, immunostaining indicated that the Ang II-induced decrease in the mean fluorescence intensity of VE-cadherin was significantly reversed by losartan and epoxomicin (Figure 6(c)). Accordingly, Ang II-induced downregulation of MKP5 and VE-cadherin protein expression and activation of p38 MAPK were also markedly reversed by losartan and epoxomicin (Figure 6(d)). These data showed that Ang II promotes LEC hyperpermeability through the AT1R-proteasome pathway *in vitro*.

### 3.7. Blocking Proteasome Activity Reduces Ang II-Induced Hypertension, Cardiac Edema, and Lymphatic Hyperpermeability

**Figure 7:**
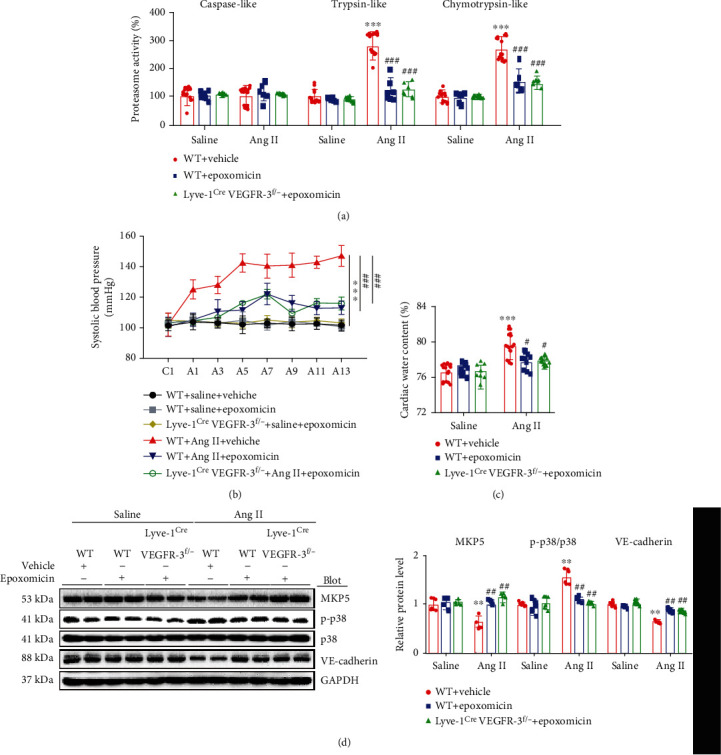
Treatment with epoxomicin attenuates the Ang II-induced increases in proteasome activity, arterial SBP, and cardiac water content and positively regulates the permeability pathway. WT and Lyve-1^Cre^ VEGFR-3^f/−^ mice were pretreated with vehicle or epoxomicin and then infused with saline or Ang II (1000 ng/kg/min) for 14 days. (a) Percentage of proteasome activity of caspase-like, trypsin-like, and chymotrypsin-like proteins in the heart (*n* = 6). (b) Average arterial SBP of mice (*n* = 6). (c) Quantitation of cardiac water content (%) (*n* = 8). (d) Immunoblot analysis of MKP5, p-p38, p38, and VE-cadherin protein levels (left) and quantification (right, *n* = 4). The data are presented as the mean ± SD; *n* represents the number of animals in each group. Statistical differences were determined by two-way ANOVA; ^∗∗^*p* < 0.01 and ^∗∗∗^*p* < 0.001 versus the WT saline+vehicle group; ^#^*p* < 0.05, ^##^*p* < 0.01, and ^###^*p* < 0.001 versus the WT Ang II+vehicle group.

To further confirm the effect of proteasome activity in regulating lymphatic permeability, WT and Lyve-1^Cre^ VEGFR-3^f/−^ mice were administered epoxomicin (0.58 mg/kg every day, a specific inhibitor of the proteasome) and infused with saline or Ang II continuously for 14 days. We found that compared with vehicle, epoxomicin obviously inhibited Ang II-induced enhancement of proteasome trypsin-like and chymotrypsin-like activity in WT and Lyve-1^Cre^ VEGFR-3^f/−^ mice (Figure 7(a)). Moreover, Ang II-induced hypertension was substantially inhibited in epoxomicin-treated WT and Lyve-1^Cre^ VEGFR-3^f/−^ mice (Figure 7(b)). Furthermore, the cardiac water content was obviously decreased in epoxomicin-treated WT and Lyve-1^Cre^ VEGFR-3^f/−^ mice compared with vehicle-treated WT controls (Figure 7(c)), indicating improvement of cardiac lymphatic function. Accordingly, the Ang II-induced reduction in MKP5 and VE-cadherin protein levels and upregulation of p-p38 MAPK expression was markedly reversed in epoxomicin-treated WT and Lyve-1^Cre^ VEGFR-3^f/−^ mice compared with vehicle-treated WT mice (Figure 7(d)).

### 3.8. Suppression of Proteasome Activity Attenuates Ang II-Induced Cardiac Dysfunction and Hypertrophic Remodeling

**Figure 8:**
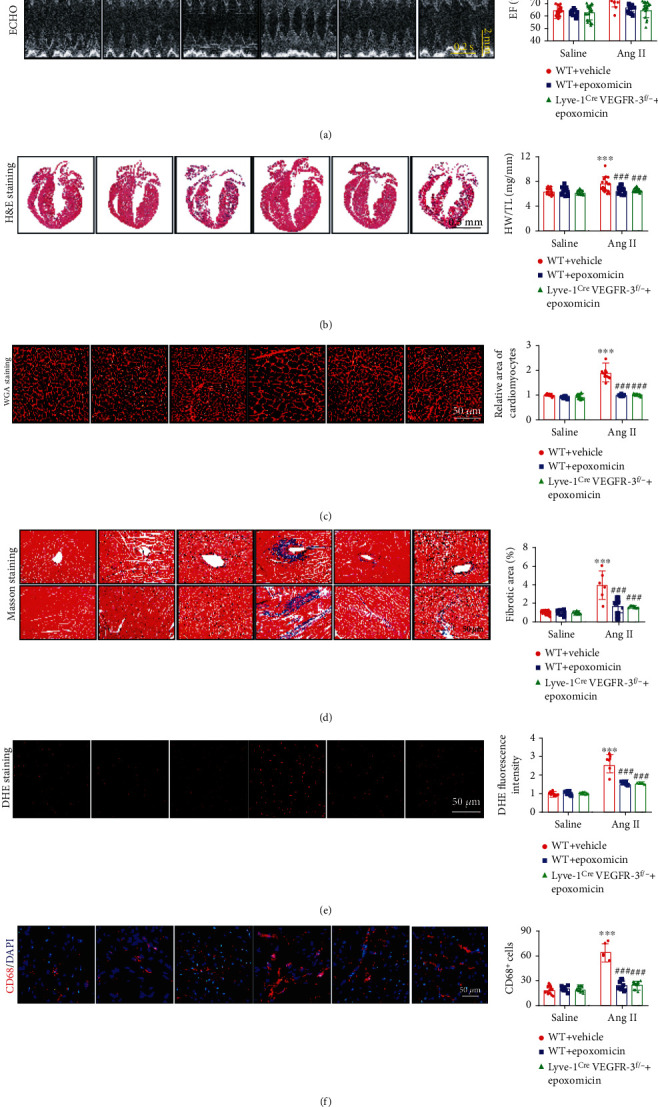
Administration of epoxomicin attenuates Ang II-induced cardiac dysfunction, hypertrophy, fibrosis, oxidative stress, and macrophage infiltration in mice. WT and Lyve-1^Cre^ VEGFR-3^f/−^ mice were pretreated with vehicle or epoxomicin and then infused with saline or Ang II (1000 ng/kg/min) for 14 days. (a) Echocardiographic analysis of the heart in M-mode (left) and analysis of EF% (right, *n* = 12). (b) Cardiac H&E staining (left) and the HW/TL ratio (right, *n* = 14). (c) Cardiac WGA staining (left) and quantitative analysis (right, *n* = 6). (d) Cardiac Masson staining (left) and quantitative analysis of the myocardial fibrosis area (right, *n* = 6). (e) Cardiac DHE fluorescence staining (red) (left, scale bar = 50 *μ*m) and quantitative analysis of relative fluorescence intensity (right, *n* = 6). (f) Cardiac CD68 immunostaining (red) and DAPI (blue) (left, scale bar = 50 *μ*m) and quantitative analysis of CD68-positive macrophages (right, *n* = 6). The data are presented as the mean ± SD; *n* represents the number of animals in each group. Statistical differences were determined by two-way ANOVA; ^∗∗∗^*p* < 0.001 versus the WT saline+vehicle group; ^###^*p* < 0.001 versus the WT Ang II+vehicle group.

We then tested whether inhibiting proteasome activity alleviates cardiac remodeling. As expected, compared with vehicle, epoxomicin markedly ameliorated Ang II-induced cardiac dysfunction, hypertrophy, and fibrosis in WT mice (as indicated by improvements in EF%, FS%, LVAW, LVPW, LVID, the E/A ratio, heart size, the HW/TL ratio, the cross-sectional area of CMs, and the fibrotic area) (Figures 8(a)–8(d), Table S3). In addition, cardiac superoxide production and infiltration of CD68^+^ macrophages were obviously decreased in epoxomicin-treated WT mice compared with vehicle-treated WT mice (Figures 8(e) and 8(f)). Similarly, the effects of Ang II were effectively attenuated in epoxomicin-treated Lyve-1^Cre^ VEGFR-3^f/−^ mice compared with vehicle controls (Figures 7(a)–7(d) and Figures 8(a)–8(f), Table S3). Therefore, these results demonstrated that epoxomicin administration inhibits Ang II-induced cardiac edema and remodeling by inhibiting MKP-5/p38 MAPK/VE-cadherin-mediated lymphatic hyperpermeability.

## 4. Discussion

**Figure 9:**
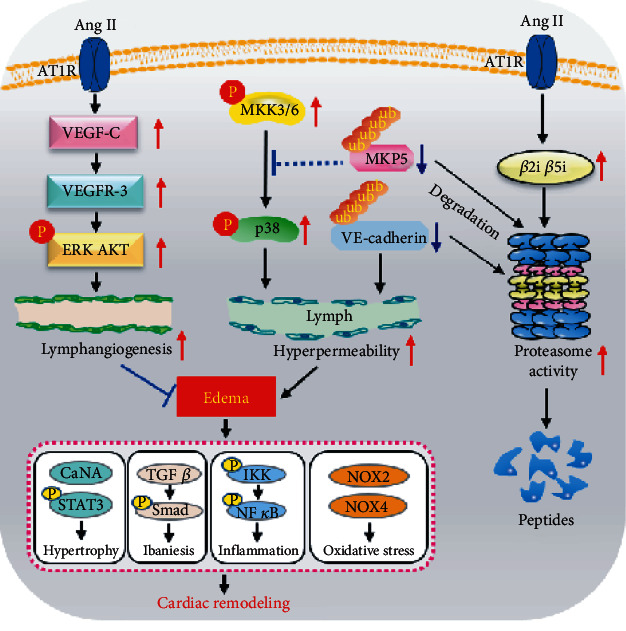
A schematic diagram showing that Ang II induces cardiac edema and hypertrophic remodeling through lymphatic-dependent mechanisms. Binding of Ang II to AT1R not only stimulates cardiac lymphangiogenesis through VEGFR-3-AKT/ERK signaling but also increases the expression and activity of the immunoproteasome catalytic subunits *β*2i and *β*5i, which promotes the degradation of MKP5 and VE-cadherin and activation of p38 MAPK, leading to lymphatic endothelial hyperpermeability and therefore a net increase in myocardial water accumulation (edema) and hypertrophic remodeling. VEGFR-3 knockdown inhibits cardiac lymphangiogenesis and aggravates cardiac edema and hypertrophic remodeling. Conversely, the proteasome inhibitor epoxomicin attenuates cardiac lymphatic hyperpermeability and hypertrophic effects.

In the current study, we provide novel evidence that both lymphangiogenesis and lymphatic vessel hyperpermeability are involved in Ang II-induced cardiac edema and hypertrophic remodeling. Knockdown of VEGFR-3 in mice inhibited cardiac lymphangiogenesis, resulting in aggravation of cardiac edema, remodeling, and dysfunction; nevertheless, treatment with the proteasome inhibitor epoxomicin reversed Ang II-induced cardiac lymphatic hyperpermeability and hypertrophic effects in mice. Mechanistically, Ang II promoted the expression and activity of the proteasome catalytic subunits *β*2i and *β*5i via AT1R, which elicited degradation of MKP5 and VE-cadherin and activation of p38 MAPK, leading to lymphatic endothelial hyperpermeability, suggesting that selective inhibition of proteasome activity may be a promising treatment for hypertension-induced cardiac disease. A schematic diagram is shown in Figure 9.

Cardiac lymphangiogenesis and function are regulated by multiple signaling pathways, including the VEGF-C-VEGFR-3 pathway [32]. VEGFR-3 is located in lymphatic and blood vessels during embryonic development but is specifically expressed on LECs during development, and complete deficiency of VEGFR-3 leads to embryonic death [33]. A previous study showed that the transcription factor NF-*κ*B can interact with Prox1 to upregulate VEGFR3 expression in LECs [34]. Our recent findings suggest that Ang II directly upregulates the expression of several lymphatic marker genes, including Prox1, LYVE-1, VEGF-C, and VEGFR-3, through AT1R, stimulating the proliferation and migration of LECs and leading to cardiac lymphangiogenesis [10]. Interestingly, numerous studies have indicated that lymphatic vessel dysfunction leads to cardiac edema, adverse remodeling, and HF during myocardial infarction or ischemia/reperfusion injury and that these effects are obviously ameliorated by stimulation of lymphangiogenesis and improvement of cardiac water homeostasis [35–39]. Recently, we further demonstrated that the VEGF-C-VEGFR-3 axis is required for cardiac lymphangiogenesis and overload-induced HF [19]. Immunoinflammatory cells and proinflammatory cytokines play an essential role in the occurrence and development of hypertension, and Ang II-induced hypertension normally results in cardiac remodeling and dysfunction [20, 40]. A published study reported that VEGFCc156s treatment significantly ameliorated Ang II-induced elevation of SBP during weeks 2 to 4 in mice and that stimulation of cardiac lymphangiogenesis relieved cardiac remodeling [41]. Indeed, our current data suggest that VEGFR-3 deficiency significantly promotes Ang II-induced hypertension, which may explain the more severe cardiac remodeling and fibrosis observed in these animals (Figure S2). Consistent with our previous data related to pressure overload-induced cardiac remodeling [19], we and another group found that the levels of VEGF-C, VEGFR-3, and cardiac lymphangiogenesis are increased in the adaptive hypertrophy phase (Ang II infusion for 2 weeks) (Figure 1) and decreased in the HF phase (Ang II infusion for 6 weeks) [41], suggesting that VEGFR-3-dependent lymphangiogenesis may be important for the transition from Ang II-induced cardiac hypertrophy to HF. Indeed, our present results show that VEGFR-3 deficiency significantly suppressed cardiac lymphangiogenesis and aggravated Ang II-induced cardiac edema, hypertrophy, fibrosis, infiltration of CD68^+^ macrophages, superoxide production, and contractile function reduction (Figures 2–4). However, activation of VEGFR-3 by VEGF-C_156S_ administration in mice increases cardiac lymphangiogenesis and ameliorates Ang II-induced cardiac edema and HF [41]. Thus, these results show that VEGFR-3 plays an essential role in Ang II-induced cardiac lymphangiogenesis, tissue edema, and hypertrophic remodeling and indicate that activation of VEGFR-3 may be a novel target for treating this disease.

The drainage performance of lymphatic vessels is influenced by not only lymphatic expansion but also LEC permeability and pumping activity, which are regulated by several factors, including VEGF-C, IL-1*β*, TNF-*α*, nitric oxide (NO), histamine, and prostaglandins [32]. Indeed, proinflammatory mediators not only affect lymphatic growth but also directly influence lymphatic integrity and permeability [32]. Numerous studies have reported that lymphatic vessel hyperpermeability is involved in various diseases, including diabetes and atherosclerosis [15, 16]. For instance, the permeability of lymphatic cells is increased in atherosclerotic Ldlr knockout mice, and apoA-I treatment significantly improves lymphatic function and abrogates atherosclerosis [15]. However, whether lymphatic vessel hyperpermeability contributes to Ang II-induced cardiac hypertrophic remodeling is still unknown. Ang II, a vasoactive peptide, activates multiple pathways by binding AT1R, and this is the essential mechanism of cardiac inflammation, oxidative stress, hypertrophy, and fibrosis, leading to hypertension and cardiac remodeling [42]. An early study showed that VEGF and Ang II can increase the permeability of the pericardium, which plays an important role in myocardial interstitial edema [43]. Importantly, our present results demonstrate that Ang II treatment significantly increased lymphatic permeability, as reflected by assessing the effect of Ang II infusion (2 weeks) on mouse hearts (the adaptive hypertrophy stage) and the impact of Ang II on mouse LEC monolayers and related signaling mediators (Figures 1(h)–1(j) and Figure 6). Ang II treatment strikingly enhanced cardiac water content and LEC permeability, probably through increasing the degradation of MKP-5 and VE-cadherin and the activation of p38 (Figures 6(c) and 6(d)). Thus, these results indicate the involvement of a new lymphatic defect, lymphatic barrier dysfunction, in Ang II-induced mouse cardiac remodeling.

There are a few dual-specificity phosphatases, including MKP-1 to MKP-26, which can dephosphorylate several pivotal signaling molecules, such as MAPKs, resulting in the regulation of their activity [30]. MKP-5 (encoded by DUSP10) can directly dephosphorylate and inactivate MAPKs, including p38 MAPK and JNK, regulating cell apoptosis, myogenesis, fibrosis, inflammation, and metabolism [30, 44–47]. Moreover, activation of p38 MAPK significantly augments the permeability of endothelial cells and reduces the expression of membrane-associated VE-cadherin [48–50]. In contrast, inhibition of p38 MAPK significantly blocks the effect of TNF-*α*-induced endothelial hyperpermeability and increases VE-cadherin expression [48]. VE-cadherin is mainly localized at the endothelial border and adhesion junctions and plays an essential role in regulating the permeability homeostasis of lymphatic and blood vessels [50]. Downregulation of VE-cadherin expression abolishes vascular endothelial cell junctions and aggravates vasal hyperpermeability [12]. Therefore, these data suggest that MKP5, p38, and VE-cadherin are crucial regulators of vascular and lymphatic vessel permeability.

Previous studies have shown that several DUSPs, including DUSP1 (MKP1), DUSP4 (MKP2), DUSP6 (MKP3), DUSP7, DUSP8 (M3/6), DUSP9 (MKP4), and DUSP16, negatively regulate ubiquitin-mediated proteasomal degradation [30]. However, whether MKP5 (DUSP10) is regulated by the proteasome remains unknown. Moreover, an early study suggested that proteasome activity is important for the degradation of VE-cadherin and that inhibition of proteasome activity by MG-132 inhibits microvascular hyperpermeability [31]. To explain how Ang II induces lymphatic vessel permeability by reducing MKP5 and VE-cadherin protein levels and activating p38 MAPK, we measured proteasome activity and examined the levels of proteasome catalytic subunits *in vivo* and *in vitro*. Consistent with previous reports in CMs, endothelial cells, and other cell types from Ang II-infused mice [26, 29, 51], Ang II treatment also significantly upregulated the expression of the immunoproteasome subunits *β*2i and *β*5i and increased trypsin-like and chymotrypsin-like activity but downregulated MKP5 and VE-cadherin expression and activated p38 MAPK in the heart and LECs (Figures 1 and 5). Conversely, treatment of mouse LECs with losartan (an AT1R antagonist) or epoxomicin (a proteasome inhibitor) markedly reversed the Ang II-induced decreases in MKP5 and VE-cadherin protein expression and the activation of p38 MAPK, leading to attenuation of lymphatic vessel hyperpermeability (Figures 5 and 6). Additionally, administration of epoxomicin led to sustained amelioration of Ang II-induced cardiac dysfunction and reductions in hypertrophy, fibrosis, superoxide levels, and infiltration of CD68^+^ macrophages accompanied by increases in MKP5 and VE-cadherin protein levels and inactivation of p38 MAPK in mice (Figures 7 and 8). Thus, the current data indicate for the first time that Ang II increases immunoproteasome activity, which promotes the degradation of MKP5 and VE-cadherin and the activation of p38 MAPK, leading to LEC hyperpermeability, cardiac edema, and hypertrophic remodeling.

The current study has several limitations: there is no direct evidence demonstrating that VEGF-C-VEGFR-3-mediated effects are specifically altered in the LECs of the intact heart; the role of MKP-5/p38 MAPK/VE-cadherin signaling in lymphatic vessel permeability during other types of hypertrophic remodeling induced by high-salt diet consumption or pressure overload and the molecular mechanism involved in the regulation of MKP-5 and VE-cadherin ubiquitination and degradation in LECs remain unclear.

## 5. Conclusion

We discovered that both cardiac lymphangiogenesis and lymphatic barrier hyperpermeability are involved in Ang II-induced adaptive hypertrophy. However, Ang II-induced hyperpermeability of LEC junctions through MKP-5/p38 MAPK/VE-cadherin signaling plays a predominant role in cardiac edema, hypertrophic remodeling, and dysfunction. Thus, selective stimulation of lymphangiogenesis and inhibition of proteasome activity may be novel therapeutic strategies for treating hypertension-induced cardiac remodeling.

## Data Availability

The data used to support the findings of this study are included in the article.

## Supplementary Materials

Supplementary Materials Figure S1: changes in the parameters of arterial systolic blood pressure, cardiac hypertrophy, and fibrosis in mice after Ang II infusion. Wild-type (WT) mice were infused with saline or Ang II (1000 ng/kg/min) for 3, 7, and 14 days. (a) Average arterial systolic blood pressure (*n* = 6). (b) qPCR analyses of myocardial atrial natriuretic factor (ANF) and brain natriuretic factor (BNP) (*n* = 6). (c) qPCR analyses of myocardial collagen I and collagen III (*n* = 6). (d) qPCR analyses of myocardial *α*-SMA (*n* = 6). Data are indicated by mean ± SD; *n* represents animal numbers of each group. Statistical difference was carried out by one-way ANOVA; ^∗^*p* < 0.05 and ^∗∗∗^*p* < 0.001 versus saline group. Figure S2: VEGFR-3 knockdown enhances Ang II-induced hypertension in mice. VEGFR-3^f/f^ and Lyve-1^Cre^ VEGFR-3^f/−^ mice were infused with saline or Ang II (1000 ng/kg/min) for 14 days. Evaluation of average arterial systolic blood pressure by the tail-cuff method (*n* = 6). Data are indicated by mean ± SD; *n* represents animal numbers of each group. Statistical difference was carried out by two-way ANOVA; ^∗∗∗^*p* < 0.001 versus VEGFR-3^f/f^+saline group; ^#^*p* < 0.05 versus VEGFR-3^f/f^+Ang II group.
