# Supplementary material for: Angiotensin II Induces Cardiac Edema and Hypertrophic Remodeling through Lymphatic-Dependent Mechanisms
Source: Oxid Med Cell Longev. 2022 Feb 18;2022:5044046. doi: 10.1155/2022/5044046 (PMC8881141; doi:10.1155/2022/5044046)
Supplement: Supplementary Materials — Figure S1: changes in the parameters of arterial systolic blood pressure, cardiac hypertrophy, and fibrosis in mice after Ang II infusion. Wild-type (WT) mice were infused with saline or Ang II (1000 ng/kg/min) for 3, 7, and 14 days. (a) Average arterial systolic blood pressure (n = 6). (b) qPCR analyses of myocardial atrial natriuretic factor (ANF) and brain natriuretic factor (BNP) (n = 6). (c) qPCR analyses of myocardial collagen I and collagen III (n = 6). (d) qPCR analyses of myocardial α-SMA (n = 6). Data are indicated by mean ± SD; n represents animal numbers of each group. Statistical difference was carried out by one-way ANOVA; ∗p < 0.05 and ∗∗∗p < 0.001 versus saline group. Figure S2: VEGFR-3 knockdown enhances Ang II-induced hypertension in mice. VEGFR-3f/f and Lyve-1Cre VEGFR-3f/− mice were infused with saline or Ang II (1000 ng/kg/min) for 14 days. Evaluation of average arterial systolic blood pressure by the tail-cuff method (n = 6). Data are indicated by mean ± SD; n represents animal numbers of each group. Statistical difference was carried out by two-way ANOVA; ∗∗∗p < 0.001 versus VEGFR-3f/f+saline group; #p < 0.05 versus VEGFR-3f/f+Ang II group. [file 5044046.f1.zip › 5044046.f1.docx]

Table S1: Primers used for quantitative real-time PCR analysis

| Gene | Forward primer (5’-3’) | Reverse primer (5’-3’) |
| --- | --- | --- |
| ANF | CACAGATCTGATGGATTTCAAGA | CCTCATCTTCTACCGGCATC |
| BNP | GAAGGTGCTGTCCCAGATGA | CCAGCAGCTGCATCTTGAAT |
| Collagen I | GAGTACTGGATCGACCCTAACCA | GACGGCTGAGTAGGGAACACA |
| Collagen III | TCCCCTGGAATCTGTGAATC | TGAGTCGAATTGGGGAGAAT |
| α-SMA | TCCTGACGCTGAAGTATCCGATA | GGCCACACGAAGCTCGTTAT |
| NOX2 | ACCGGGTTTATGATATTCCACCT | GATTTCGACAGACTGGCAAGA |
| NOX4 | CAGATGTTGGGGCTAGGATTG | GAGTGTTCGGCACATGGGTA |
| VEGFR-3 | CCGCAAGTGCATTCACAGAG | TCGGACATAGTCGGGGTCTT |
| IL-1β | TGCCACCTTTTGACAGTGATG | TGATGTGCTGCTGCGAGATT |
| IL-6 | TGATGGATGCTACCAAACTGGA | TGTGACTCCAGCTTATCTCTTGG |
| β1i | CTGGAGCTACACGGGTTGGA | ATATACCTGTCCCCCCTCACATT |
| β2i | CAGCCGTCTGCCCTTTACTG | AGAGCCCAGGTCACTCAGGAT |
| β5i | CTTGGCACCATGTCTGGTTGT | CCGGTACTGCAGCATCATGT |
| GAPDH | GGTTGTCTCCTGCGACTTCA | GGTGGTCCAGGGTTTCTTACTC |

ANF, atrial natriuretic factor; BNP, brain natriuretic factor; α-SMA, α-smooth muscle actin; NOX2, NADPH oxidase 2; NOX4, NADPH oxidase 4; VEGFR-3, vascular endothelial growth factor receptor 3; IL-1β, interleukin 1 beta; IL-6, interleukin 6; β1i, proteasome subunit beta-1i; β2i, proteasome subunit beta-2i; β5i, proteasome subunit beta-5i; GAPDH, glyceraldehyde 3-phosphate dehydrogenase.
