# Supplementary material for: Angiotensin II Induces Cardiac Edema and Hypertrophic Remodeling through Lymphatic-Dependent Mechanisms
Source: Oxid Med Cell Longev. 2022 Feb 18;2022:5044046. doi: 10.1155/2022/5044046 (PMC8881141; doi:10.1155/2022/5044046)
Supplement: Supplementary Materials — Figure S1: changes in the parameters of arterial systolic blood pressure, cardiac hypertrophy, and fibrosis in mice after Ang II infusion. Wild-type (WT) mice were infused with saline or Ang II (1000 ng/kg/min) for 3, 7, and 14 days. (a) Average arterial systolic blood pressure (n = 6). (b) qPCR analyses of myocardial atrial natriuretic factor (ANF) and brain natriuretic factor (BNP) (n = 6). (c) qPCR analyses of myocardial collagen I and collagen III (n = 6). (d) qPCR analyses of myocardial α-SMA (n = 6). Data are indicated by mean ± SD; n represents animal numbers of each group. Statistical difference was carried out by one-way ANOVA; ∗p < 0.05 and ∗∗∗p < 0.001 versus saline group. Figure S2: VEGFR-3 knockdown enhances Ang II-induced hypertension in mice. VEGFR-3f/f and Lyve-1Cre VEGFR-3f/− mice were infused with saline or Ang II (1000 ng/kg/min) for 14 days. Evaluation of average arterial systolic blood pressure by the tail-cuff method (n = 6). Data are indicated by mean ± SD; n represents animal numbers of each group. Statistical difference was carried out by two-way ANOVA; ∗∗∗p < 0.001 versus VEGFR-3f/f+saline group; #p < 0.05 versus VEGFR-3f/f+Ang II group. [file 5044046.f1.zip › 5044046.f2.docx]

Table S2: Echocardiographic parameters of wild-type (VEGFR-3^f/f^) and VEGFR-3 knockout (Lyve-1^Cre^ VEGFR-3^f/−^) mice after Saline or Angiotensin II (Ang II) infusion for 14 days

|  | **Saline** | | **Ang II** | |
| --- | --- | --- | --- | --- |
|  | **VEGFR-3^f/f^** | **Lyve-1^Cre^ VEGFR-3^f/−^** | **VEGFR-3^f/f^** | **Lyve-1^Cre^ VEGFR-3^f/−^** |
| EF (%) | 64.02±5.37 | 62.84±5.37 | 76.40±4.01^**^ | 45.50±12.41^###^ |
| FS (%) | 33.90±3.70 | 33.05±3.28 | 44.06±3.52^***^ | 22.60±7.32^###^ |
| LVAW; d(mm) | 0.82±0.11 | 0.85±0.14 | 1.11±0.14^***^ | 0.67±0.12^###^ |
| LVPW; d(mm) | 0.79±0.16 | 0.80±0.09 | 1.01±0.15^*^ | 0.66±0.12^###^ |
| LVID; d(mm) | 3.39±0.25 | 3.43±0.25 | 2.90±0.28^**^ | 3.85±0.29^###^ |
| LVAW; s(mm) | 1.27±0.11 | 1.38±0.16 | 1.64±0.12^***^ | 1.13±0.12^###^ |
| LVPW; s(mm) | 1.15±0.17 | 1.08±0.12 | 1.39±0.15^*^ | 1.00±0.17^###^ |
| LVID; s(mm) | 2.24±0.23 | 2.42±0.32 | 1.79±0.23^*^ | 2.82±0.46^###^ |
| E (mm/s) | 526±18 | 532±25 | 526±24 | 473±32^###^ |
| A (mm/s) | 295±30 | 296±29 | 461±28^***^ | 543±40^###^ |
| E/A Ratio | 1.81±0.21 | 1.82±0.25 | 1.15±0.09^***^ | 0.87±0.07^#^ |

Values: means ± SD (n = 10);

^*^*p*<0.05, ^**^*p*<0.01 and ^***^*p*<0.001 vs. VEGFR-3^f/f^ + Saline; ^#^*p*<0.05 and ^###^*p*<0.001 vs. VEGFR-3^f/f^ + Ang II; EF, ejection fraction; FS, fractional shortening; LVAW; d, left ventricular anterior wall at end-diastole; LVPW; d, left ventricular posterior wall at end-diastole; LVID; d, left ventricular internal dimension at end-diastole; LVAW; s, left ventricular anterior wall at end-systole; LVPW; s, left ventricular posterior wall at end-systole; LVID; s, left ventricular internal dimension at end-systole; E, peak early transmitral inflow mitral E velocity; A, transmitral inflow velocity due to atrial contraction.

Table S3: Echocardiographic parameters of wild-type (WT) mice for 14 days of Saline or Angiotensin II (Ang II) infusion and co-treated with vehicle or epoxomicin

|  | **Saline** | |  | **Ang II** | |  |
| --- | --- | --- | --- | --- | --- | --- |
|  | **WT+ Vehicle** | **WT+ Epoxomicin** | **Lyve-1^Cre^ VEGFR-3^f/−^ + Epoxomicin** | **WT+ Vehicle** | **WT+ Epoxomicin** | **Lyve-1^Cre^ VEGFR-3^f/−^ + Epoxomicin** |
| EF (%) | 64.61±3.80 | 63.05±9.84 | 63.18±11.63 | 75.88±14.44^***^ | 65.39±13.38^###^ | 65.85±13.97^###^ |
| FS (%) | 34.41±2.86 | 33.26±5.26 | 33.61±6.43 | 43.72±9.53^***^ | 34.27±8.20^###^ | 34.25±8.42^###^ |
| LVAW; d(mm) | 0.70±0.07 | 0.71±0.11 | 0.72±0.13 | 0.95±0.21^***^ | 0.74±0.18^###^ | 0.75±0.17^###^ |
| LVPW; d(mm) | 0.63±0.06 | 0.64±0.12 | 0.64±0.12 | 0.91±0.21^***^ | 0.73±0.18^###^ | 0.70±0.17^###^ |
| LVID; d(mm) | 3.34±0.28 | 3.40±0.56 | 3.47±0.63 | 2.90±0.63^**^ | 3.28±0.66^#^ | 3.32±0.68^##^ |
| LVAW; s(mm) | 1.17±0.09 | 1.20±0.20 | 1.13±0.23 | 1.58±0.34^***^ | 1.25±0.29^###^ | 1.21±0.29^###^ |
| LVPW; s(mm) | 0.98±0.10 | 0.99±0.17 | 0.93±0.18 | 1.43±0.32^***^ | 1.03±0.27^###^ | 1.02±0.26^###^ |
| LVID; s(mm) | 2.19±0.17 | 2.27±0.37 | 2.43±0.46 | 1.64±0.49^***^ | 1.96±0.45^##^ | 2.36±0.51^###^ |
| E (mm/s) | 536±54 | 521±83 | 528±97 | 541±102 | 540±103 | 534±111 |
| A (mm/s) | 295±44 | 280±50 | 291±55 | 489±122^***^ | 310±96^###^ | 312±92^###^ |
| E/A Ratio | 1.84±0.17 | 1.87±0.31 | 1.82±0.35 | 1.11±0.46^***^ | 1.75±0.41^###^ | 1.72±0.42^###^ |

Values: means ± SD (n = 12);

^**^*p*<0.01 and ^***^*p*<0.001 vs. WT Saline + Vehicle; ^#^*p*<0.05, ^##^*p*<0.01 and ^###^*p*<0.001 vs. WT Ang II + Vehicle; EF, ejection fraction; FS, fractional shortening; LVAW; d, left ventricular anterior wall at end-diastole; LVPW; d, left ventricular posterior wall at end-diastole; LVID; d, left ventricular internal dimension at end-diastole; LVAW; s, left ventricular anterior wall at end-systole; LVPW; s, left ventricular posterior wall at end-systole; LVID; s, left ventricular internal dimension at end-systole; E, peak early transmitral inflow mitral E velocity; A, transmitral inflow velocity due to atrial contraction.
